
JOURNAL INFORMATION
==============================
NLM Title Abbreviation: Atlanta Med Surg J (1884)
NLM Journal ID: 101251623

Atlanta Medical and Surgical Journal (1884)

Publisher: Biomedical Journal Digitization Project

ARTICLE INFORMATION
==============================
PMCID: PMC9201858
PMID: 35829608
Article version: 1
Subjects: Selections and Abstracts

Selections and Abstracts

Print publication date: 1899 Feb
Volume: 15
Volume Series: New Ser
Issue: 12
First page: 847
Last page: 864
License: This work is free of known copyright restrictions. For more information, please see PMC Back Issue Digitization.
License URL: https://creativecommons.org/publicdomain/mark/1.0/

==============================



### SELECTIONS AND ABSTRACTS.

### Quinin in Malarial Hemoglobinuria.

From time to time we read articles in which the assertion is made that the administration of quinin during the existence of hemoglobinuria in malarial infection aggravates the symptom. In fact, it is held by some writers and practitioners that quinin will produce this condition in malarial fever. While this view is sustained by some who practice medicine in malarial districts, it can be positively stated that the vast majority of practitioners in those districts do not hold to any such belief.

As we have increased our knowledge concerning the nature of malarial fever and the plasmodium which produces it, to that extent have these vagaries once propounded and upheld been refuted. At a time when malarial fever was believed to be due to carbonic acid, organic material, effluvia, paludism, and various other theories devoid of proof, and at a time when bacteriology and pathology had not been established upon irrefragible evidence, many plausible theories were set up only to be dissipated by the scathing light of definite knowledge established by the study of these two branches of medicine. Bacteriology has taken malarial fever from the category of diseases of unknown cause and placed it forever among the infective diseases. Pathology has shown us the condition existing during the life of the patient.

In malarial fever we have come to study the internal organs and we find that the spleen is enlarged, soft and congested, the blood-vessels being filled with red-blood cells in all stages of disintegration, leucocytes, blood pigment and the plasmodium in various stages of development. In the liver, congestion is the rule, producing a stagnation of the portal circulation and a deficiency of bile, while the kidneys may be enlarged, but in most cases show no evidences of parenchymatous or interstitial change.

In the kidney of malarial hemoglobinuria, Thayer, Pellarin, Kierner and Kelsch have shown a condition bordering on a parenchymatous degeneration with interstitial hemorrhages and blood corpuscles in the tubules, yet this condition is frequently found in other conditions than malarial fever and in which quinin may be used with impunity without producing hemoglobinuria.

It should be remembered that it is not alone from the study of the internal organs that our knowledge has been obtained regarding this subject, for the plasmodium has become more bold and exhibits itself in the peripheral circulation. Here then its cycle can be observed and studied—first is the small hyaline segment, with a rotatory twisting and branching movement, next, as the flattened yellowish or hyaline cell of many sizes and shapes, and last, as the full-grown, vigorous organism containing fine masses of hemoglobin, which has been derived from the red-blood cell set free by the necrobiotic process engendered by its toxin.

With each paroxysm there is set free in the circulating medium more hemoglobin, which must be stored in the organs whose duty it is to receive it. The internal viscera have no choice but to receive its repository and when it is thrown off they receive the legacy of its coloring agents which remain in the intracellular tissue. Briefly, this may be said to be a summary of the morbid anatomy of malarial fever, which also includes hemoglobinuria of malarial origin, which must be considered a symptom and not a distinct entity. It should also be remembered that the bright red urine observed is not always a hematuria. In fact, a hematuria is the rare exception, for hemorrhages, of whatever nature, are uncommon in all varieties of malarial fever. It is the blood coloring matter, and not the red-blood corpuscles, which produces the glow in the cheek in the fastigium of the fever, and its absence which denotes the pallor of a cachexia. But in the later stages of a congested kidney blood corpuscles may be present.

Why the hemoglobinuria of malarial origin has been singled out among all the other varieties and stated to be increased by the use of quinin, is not clear. Thus we have: 1, paroxysmal hemoglobinuria, and 2, toxic hemoglobinuria, including that due to chlorate of potash, carbolic acid, naphthol, carbon dioxid, and the poisons of infectious fevers, yellow fever, scarlet fever, typhus fever and malarial fever.

The rules of logic by which some endeavor to prove the sequence of events, from the administration of quinin to the production of hemoglobinuria, are not circumscribed within the boundary of therapeutics; no such proof exists. Preponderating evidence plainly shows that quinin in the largest doses will neither lead to a hematuria nor a hemoglobinuria. It neither causes a necrobiosis of the red-blood corpuscles, nor causes a congestion of the kidneys. Thus, Baillio reports two soldiers who took three drams of quinin, producing, in both cases, cramps in the epigastric region, accompanied by vomiting, paleness of face, dilated pupils, irregular, slow pulse, ringing of the ears, syncope, and death in one case, while in the other the symptoms gradually disappeared. Briquet reports a case in which a man took fifty-five drams of the salt without the production of hemoglobinuria or hematuria, and Guersent has observed a woman who became deaf, dumb and blind after having taken ten drams of quinin sulphate within a few days.

The foremost physicians who practice in malarial districts are not afraid of quinin. If four grains of quinin every four hours will not break up a chill, ten and fifteen grains are given with impunity and without producing a hemoglobinuria; and if fifteen will not suffice in a case of pernicious fever, thirty grains are frequently given hypodermically.

The exact chemic composition of the toxin produced by the plasmodium malariæ is unknown. That it is actively poisonous is beyond doubt. Thus a small amount of blood from a patient suffering with malarial fever has been obtained and transfused into another patient, with the result that a malarial fever has been produced. The urine of a patient suffering from malarial fever has been found to be more toxic immediately after the paroxysm than before it. The rupture of the enveloping membrane of the affected red-blood corpuscle marks the onset of the paroxysm and the chill. Fever is only the reaction of the system to expel the poisonous material.

Having, therefore, the two factors, quinin and toxin, in the blood concurrently, which one will be most patent in the production of hemoglobinuria? The toxin of malaria causes a hypertrophy of the spleen, which will destroy the red-blood globules, will cause a chill, and being poisonous, may irritate the kidneys, while quinin has never been proven to be the etiologic factor in any of these conditions, and, therefore, must be the least causal agent in the production of a symptom for which it should be used.—*Journal of the American Medical Association.*

---

### TREATMENT OF TETANUS BY MEANS OF INTRACEREBRAL INJECTION OF ANTITOXIN.

In an editorial abstract which appeared in the *Journal* a few weeks ago, reference was made to the interesting story of Roux and Borrel concerning the relation of tetanus toxin to nerve-cells. It will be recalled that when the toxin is injected directly into the brain a peculiar form of tetanus, which Roux and Borrel call cerebral tetanus, results. The symptoms are characterized by psychic excitement, intermittent convulsions, motor disturbances and polyuria, and depend upon the fixation of the ganglion cells. It will also be remembered that Wasserman and Takaki were the first to make experiments that called the attention of investigators to this peculiar affinity of the tetanus toxin for the nerve-cells, inasmuch as they showed that the same intimate union between toxin and nerve-cells occurs when bits of nerve substance and toxin are mixed together outside the body. Roux and Borrel also showed that injection of antitoxin into animals affected with cerebral tetanus has no effect, because the antitoxin does not have the same affinity for the nerve-cells as the toxin, does not therefore come in contact with the poison, and proves entirely powerless against the poison that has already been fixed by the nerve-cells. This they considered to be one of the principal reasons why, in man as well as in animals, subcutaneous or intravenous injections of antitoxin in tetanus so often fail. The antitoxin may, of course, neutralize the toxin circulating in the blood, but in order to accomplish most, the antitoxin should be placed where the toxin is acting; of forty-five tetanized guinea-pigs treated with intracerebral injections of antitoxin, thirty-five recovered, whereas seventeen treated with subcutaneous antitoxin injections all died, excepting two. Theory and experiments seemed to agree beautifully.

The striking results obtained in animals have justified the intracerebral injection of antitoxin in man. Inasmuch as it may be considered that this form of treatment would afford the patient the only chance possible, he should have the benefit of it, although it might be objected that the risks connected with this treatment are numerous.

In an article containing a *résumé* of the reports of cases treated by means of intracerebral injections of tetanus antitoxin, Rambaud* finds that the risks are in reality trivial. If the intracerebral injection is made in a neutral area, as for instance, in the frontal lobes, the results of the trauma are reduced to the minimum. Under all circumstances the injury caused by the insertion of a fine hypodermic needle into the brain is trifling; the quantity of serum introduced is small, and when introduced slowly no undue compression needs to be caused. Hemorrhage from the pia and dura can be readily avoided, and the button of bone removed being preferably small, healing takes place rapidly. At the same time as intracerebral injections are the most effecient mode of treatment, it is nevertheless necessary, for the reasons already alluded to, to employ intravenous and subcutaneous injections of antitoxin, in order to neutralize the toxins circulating in the blood and that which is continuously being secreted at the site of the injury.

The first human case treated in this manner occurred only two months after the publication of Roux and Borrel's researches. Of the twelve cases which have now been treated by this plan, three have occurred in this country, the first being one published in the *New York Medical Journal* for December 17, 1898, by Charles A. Church, of Passaic, N. J. Recovery took place. Rambaud refers to two more cases, one of which died from tetanus. In the other of these cases, in which the tetanus developed ten days after a laparotomy, the tetanic symptoms subsided after intracerebral and subcutaneous injections of antitoxin, but death resulted, apparently from anemia, nephritis and sepsis, the fatal issue taking place eleven days after the complete disappearance of the tetanic mani-

*New York Medical Journal, December 17, 1898.

festations, which were of the bulbar type of tetanus, and consequently, according to Roux and Borrel's teaching, of bad prognostic omen. In these two cases, one may therefore be said to have recovered under this mode of treatment.

The most delicate part of the operation in intracranial injection is the injection itself. It is necessary to have a very perfect syringe, which must be absolutely aseptic. In order to make it possible to inject the antitoxin slowly, drop by drop, Rambaud has constructed a syringe which holds 3 c.c., the rod of which fits a thread inside the cap, so that the injection can be made by a screwing motion of the thumb-piece. The head of the needle is of conic shape, in order to fit the trephine hole made in the skull. The trephine used in Bambaud's cases had a crown whose diameter was about 7 mm. The cone-shaped head of the needle makes it possible to firmly fix the needle in the trephine hole; between the needle and the syringe is a flexible rubber tube connection. With this arrangement there is no risk of the needle moving after it has been properly inserted. The injection should be made in from ten to twelve minutes. The cone of the needle is provided with a groove so as to afford passage to any fluid which might otherwise cause compression.

It would seem that this mode of treatment of tetanus, the mortality from which is yet very large, even when treated promptly by means of the ordinary subcutaneous or intravenous injections of antitoxin, would merit an extended trial. The actual results of the intracerebral injections of antitoxin can only be fully determined when a large number of cases have accumulated. It may also be allowed to indicate that in all probability a single intracerebral injection of tetanus antitoxin will not always suffice to neutralize the toxin fixed in ganglion cells, scattered, perhaps, over a large part of the brain. It may be found neccessary to make several injections into the brain in various places.—*Journal of the American Medical Association.*

---

### PNEUMONIA IN CHILDREN.

Pneumonia in childhood has of late been deeply occupying the attention of pediatrists, and without doubt much good has been effected by the interchange of opinions, and by the ventilation of the subject generally. At the meeting called together by the chairman of the pediatric section of the New York Academy of Medicine, many points in connection with the treatment of the disease were made clear which, up to that time, had been decidedly misty. When the fact is considered that pneumonia is one of the most deadly maladies to which children are prone, it cannot be denied that the time had come for a certain consensus of opinion to be arrived at in regard to its treatment. But before touching on the subject of treatment, it may be as well to refer briefly to the light that has been thrown on the etiology of the disease by means of the researches of bacteriologists. This phase of the matter has been extremely well set forth in an article contributed to the Edinburgh *Journal* of August 26th, by Dr. James Carmichael, physician to the Edinburgh Royal Hospital for Sick Children, who says:

"Bacteriology has contributed largely to our knowledge of the disease. The rôle which micro-organisms play in the disease is certainly a very important one, as without doubt the toxemia resulting from their growth and development would appear to be not only the main factor in the production of the constitutional symptoms, but often the principal cause of danger to life. Recent bacteriological investigation shows that quite a number of organisms are found associated with this disease. Netter gives the bacteriology in forty-two cases—in twenty-five primary cases. Friedlander's capsuled coccus or pneumobacillus was found in ten, streptococcus in eight, staphylococcus in five, Fränkel's capsuled coccus in two. In seventeen of the cases there was mixed infection thus: In five pneumococcus and streptococcus, five streptococcus and staphlyococcus, three streptococcus and capsuled coccus, two pneumococcus, streptococcus and staphlyococcus, one pneumococcus and capsuled coccus. Mosny and Netter both agree that in cases marked by lobular distribution, streptococcus was usually met with alone or with pneumococcus, whereas those in which the distribution was lobar, catarrhal and fibrinous variety combined, one or other of the forms of pneumococcus, either Fränkel's or Friedlander's organisms, were generally found alone. Other investigators who have studied the different forms of pneumonia have arrived at the conclusion that it is no one organism that causes the disease, but two and sometimes more. Thus plainly proving that the pneumonia of childhood cannot be regarded as a specific disease, seeing that various organisms produce the pathological changes in the lung tissues."

The foregoing may be said to be a short, up-to-date presentment of the germ theory of pneumonia in children, and would appear in the main correct. The next forward step, then, is to discover a really reliable antitoxin, but until this is done we must ourselves sift the different modes of treatment at present practiced, and learn from the experience of others in order to arrive at a satisfactory conclusion. At the meeting of the pediatric section, referred to above, some definite opinions were expressed, and from the views of the majority of the speakers we can form our own judgment as to the most beneficial and widely practiced methods of treatment. For example, it was agreed on all hands that rest and complete isolation is absolutely necessary; that careful nursing is one of the most important considerations, and the opinion was unanimous that effective ventilation is essentual. The meeting was opposed, on the whole, to the active use of drugs, and few voices where raised in favor of stimulants, while hot poultices were condemned *in toto.* On the question of bathing, opinions differed most widely. Not, however, so far as the beneficial effects of baths themselves are concerned, but with respect to their temperature. Some advocated baths as high as 100 F.; others deemed 70 F. a heat better calculated to produce the wished-for results, while one speaker advised the employment of the wet-sheet, cooled by ice. The large majority of the speakers deprecated extremes of either heat or cold, but recommended the adoption of a middle course.

To refer again to Dr. Carmichael's article. In very severe cases, with much cyanosis, he suggests the inhalation of oxygen, the small patient lying under a tent, and states that in many cases he has known the treatment to be of signal benefit. In infantile pneumonia, many physicians in Great Britain assert that belladona acts like a charm. There would, however, seem to be some diversity of views in this country and Great Britain as to the efficacy of the drug treatment of pneumonia in children. The judgment of medical men here, as evidenced by the expression of their opinions at the meeting of the pediatric section, is distinctly against active medication. In Great Britain, on the other hand, the conservative treatment still, to a large extent, holds sway. As, however, the study of children's diseases as a specialty has been more closely and for a far longer period pursued in America than in the United Kingdom, we may claim—without laying ourselves open to the charge of undue presumption—to be more fitted to pass an authoritative opinion on the matter. The pediatric section deserves the thanks of every medical man for the able manner in which the whole subject was placed before them.—*Pediatrics.*

---

### THE EXCISION OF CARBUNCLES.

Mr. Rushton Parker (*British Medical Journal,* November 26th) again calls attention to the subject of the complete extirpation of carbuncles. The ordinary methods of palliative expectation and excision, in his opinion, in which Sir James Paget in his *Clinical Lectures* seems to concur, show no advantage of one course over the other. The two principal features of carbuncles are (1) pain and (2) liability to septic poisoning. Incision, he asserts, does not relieve the pain, and does nothing to prevent or arrest septic poisoning. It is a severe operation, requiring an anesthetic, and is itself liable to be followed by septic poisoning through newly opened channels.

The case is quite different, he continues, with extirpation, which, however extensive, is not a much more severe operation than incision. But what a contrast in the result! Ask a patient the day after extirpation what he thinks of it, and he replies, "I feel no pain whatever, only a little weak"; "I feel as if a load had been taken off me." The pain resided in the inflamed, stretched, and infiltrated integuments, which in thorough extirpation are all removed, leaving only a wound in healthy tissues. The same applies to septic poisoning, which at first is generally quite local and confined to the affected tissues. When these are removed the poisonous focus has gone.

The extirpation of carbuncle by excision and scraping, according to the texture of the affected tissues, is just as rational as the excision of tuberculous glands, the scraping of lupus, and the removal of sloughs, while experience amply proves its value. Among the twelve cases hitherto reported by him, two were in women, situated on the face; the rest were in men. In one or two cases the relief to pain was not complete, and this he attributes to insufficient thoroughness in the excision. If the carbuncle is very soft there may be a temptation to rely on thorough scraping alone, and so to leave some of the disease behind. It is advisable to remove all, and to thoroughly cut away with knife or scissors what can not be scraped.

He then reports five more cases, of which one proved fatal. This he attributes to not critically watching the sore to detect any reappearance of the carbuncular process, and not removing thoroughly all recognizable diseased parts. Diseased spots he touches with pure carbolic acid which effectually renders them aseptic and is at the same time anodyne.—*New York Medical Journal.*

---

### ANTISTREPTOCOCCUS SERUM IN CHANCROID.

Mr. James Moore, F. R. C. S. Edin. (*British Medical Journal,* November 26th), states that during the past eight months he has used antistreptococcus serum in forty-eight cases of acute inflammatory bubo, and in only seven cases did suppuration occur. He records two very interesting cases. From his experience with antistreptococcus serum in venereal sores and their complications, he has arrived at the following conclusions:

1. While recognizing the great importance of early local antiseptic treatment of the chancroid, he believes that if five cubic centimeters of the serum are injected subcutaneously into each inguinal region in cases in which inflammatory bubo is likely to develop, it will prove a good prophylactic measure, and assist in healing the chancroid.

2. If inflammatory bubo has already developed, and the acute inflammatory symptoms have not been present more than forty-eight hours, ten cubic centimeters injected into the inguinal region corresponding to the inflamed gland will cause resolution in the majority of cases.

3. If there is evidence of pus formation the serum may possibly limit the extension of the suppuration, but in this class of cases his results have been anything but satisfactory.

4. The serum should always be injected into the area drained by the infected gland, preferably the right and left inguinal region. He has not seen good results by injecting it into remote areas.

5. In phagedenic ulceration complicating venereal sores this serum would appear not only to neutralize the toxins in the blood, but also to bring about a healthy condition of the ulcer.—*New York Medical Journal.*

---

### FARADAISM IN DILATATION OF THE OS UTERI.

Dr. Jeannie W. Martine (*Journal of Electrotherapeutics*, December) describes a dilator, similar to Nott's dilator, which is connected with one pole of a faradaic battery by one terminal, and by another with a large pad which is placed on the abdomen. The anesthetic effect of the fine wire faradaic coil are, according to the author, of material assistance.

Dr. Martine says :

"I have used this instrument in a variety of cases. I wish to mention one class where the physician does not, as a rule, consider divulsion necessary. I mean in subinvolution complicated with endometritis; here divulsion causes, first, drainage. Secondly, it acts as massage and sets up a better circulation. The electricity helps us here by its stimulating properties more than its anesthetic effects, as the uterus is rarely sensitive in subinvolution. My electrical dilator is one of my best friends in nearly all cases of uterine disease, and it can, of course, be used with the galvanic as well as faradaic currents, and I have yet to see a case so sensitive that I could not dilate by its judicious use. It is indeed simply wonderful how the long, fine wire coil of an Engleman faradaic battery will benumb the parts so that little or no distress is felt. I, however, give myself plenty of time in treating sensitive subjects and only do very little at each treatment. One important point I wish to mention: The anesthetic effect of electricity, faradaism at least, is only felt while the current is turned on, and this is the objection to using graduated sounds, much pain being caused by withdrawing, turning off current, and reintroducing a larger size. Theoretically why this is the case I cannot say; practically I know it to be so; but the instrument I have described allows current to flow while we are dilating and overcomes that difficulty."

This procedure might be worth a trial in stricture of the urethra, though the catalytic properties of the faradaic current are slight as compared with those of galvanism.—*New York Medical Journal.*

---

### Solomon Right Again.

The *India Lancet* for November 16th quotes the following:

"In *Notes and Queries,*" says the *New York Times,* "there is a story quoted written by Mir Muhammad M'asum in 1660. An exiled prince meets a man who is carrying what are designated as 'hakku tubes.' Looking at the man, the prince discovers that just as long as the man holds the hakku tubes his entire interior economy is visible. The prince at once purchases the tubes. Coming to Ghuzni, he finds a patient. The King of Ghuzni is suffering from dyspepsia. There is good reason for the king feeling so uncomfortable, for when the hakku tubing is applied, it is discovered that the patient had swallowed not less than two water snakes. Readers will then please observe that the X-rays have been anticipated. The original of the story is found in Elliot's *History of India* by its own historians. After a while we may discover that Noah's ark had water-tight bulkheads and twin screws."—*New York Medical Journal.*

---

Muscular Atrophies of Hysterical Origin. (H. DuBois-These de Paris. Five Original Observations. *Revue Neurologique,* October 30, 1898.)

Muscular atrophies of hysterical origin frequently follow a hemiplegia or traumatism, but they may appear without apparent cause. They are simple atrophies. They usually affect the muscles generally, but may attack particular groups. They begin abruptly and reach their maximum condition with great rapidity, remaining stationary for a time and gradually disappearing with the disappearance of the phenomena which have provoked them (paralysis, contractures, etc.)

They are always accompanied by hysterical symptoms, especially hemianesthesia, anesthesia of ocular and pharyngeal mucous membranes and the hysterical pupil. They are sometimes accompanied by fibrillary tremors. The RD does not appear in the most marked cases.—*Riggs.*

---

### LOCAL ANESTHESIA.

Dr. Bagot (*Journal des practiciens*) considers that the tonic action of sparteine on the heart combats the depressant effect of cocain, while aiding the local anesthestic effect of the latter. He recommends the following in each powder:

| | |
|---|---|
| Hydrochloride of cocain | ½ grain. |
| Sulphate of sparteine | ¾ grain. |

For use, dissolve one powder in either fifteen or twenty drops of boiled water at the time of injection. Begin with the weaker solution. Fifteen drops are injected in the line of operation on one side of the small tumor to be removed; after waiting seven or eight minutes, the other half on the other side. At the end of some moments he commences the operation on the first side injected, and by the time that is finished anesthesia is complete on the other side.—*New York Medical Journal.*

---

### A TROPICAL SCHOOL OF MEDICINE IN LONDON.

Our usually accurate contemporary, the *Philadelphia Medical Journal,* in its issue for December 24th, has a paragraph with this heading. London, we are informed, is a pretty "hot" place—in fact quite "sultry" in some respects; but for all that, it is some degrees north of the Tropic of Cancer. A School of Tropical Medicine is, however, what we believe our contemporary to mean.—*New York Medical Journal.*

### THE USES AND EFFECTS OF MANGANIFEROUS IRON PEPTONE.

BY DR. JULIUS HEITZMANN, VIENNA.

The employment of iron preparations both in essential anemia (chlorosis) and in the symptomatic forms of this affection produced by severe losses of blood, dates from the earliest times. Long before the chemical relation of this effect was known, these remedies were administered on the ground of pure empirical experience.

When Hannon pointed out the high significance of manganese, as well as of iron, with regard to the absorption of oxygen by the blood, and when this discovery was confirmed by Ruehle, efforts were made to produce, by combination of both remedies, preparations which would best fulfill the therapeutic indications in all directions.

Former attempts of this kind failed to give the desired results. The aim was to combine both metals in such a form as would enable them to be absorbed throughout the entire extent of the alimentary canal, and at the same time be devoid of disagreeable taste which would prevent their prolonged administration. After a series of experiments made in this direction I found in the preparation discovered by Dr. A. Gude (Pepto-Mangan—Gude) a remedy which fulfilled the above requisites, and can recommend it most heartily.

Pepto-Mangan—Gude is a clear, dark, wine-red fluid, having an agreeable, non-metallic, astringent taste. The latter property gives it a great advantage over other similar preparations, for the remedy is always taken with pleasure, and may therefore be administered for a long time without exciting the disgust of the patient. No irritation of the stomach is produced, nor is the digestion disturbed in the least respect; indeed, as regards the latter, a stimulation of the long-absent appetite could be demonstrated within a short time.

The Pepto-Mangan—Gude, usually mixed with some water, is prescribed in doses of two or three dessertspoonfuls, increased to as many tablespoonfuls per day. An especially agreeable manner of administration is by addition of cold milk, which then assumes a light chocolate color and agreeable taste. Prescribed in this form we obtain from this preparation everything that could be expected from a remedy for anemia. The Pepto-Mangan—Gude may also be mixed with white and sweet wines, excepting the red wines which contain tannic acid, and an occasional change in the manner of administration is sometimes of advantage, especially in the case of children.

The diet, during the use of this preparation, should consist of milk, meats—especially ham—fowl, soft-boiled eggs, and other easily digested foods. On the other hand, sour and fatty foods, red wines and raw fruits are to be avoided.

The remedy is to be administered for a number of weeks, especially in cases of chlorosis, but in the case of young girls up to 12 years of age it is best to commence with a daily dose of two teaspoonfuls (ten grammes). In adults the dose of the Pepto-Mangan—Gude may be increased in a few days to one tablespoonful twice or thrice daily, or even to ten or twenty grammes. The preparation should be well protected from the light, and preserved in a cool place in a well-stoppered bottle.

I have employed the Pepto-Mangan—Gude with much success both in chlorosis and in cases of anemia in girls and women due to loss of blood, menorrhagia, metrorrhagia, inflammation of the pelvic organs, peri- and parametritis, or prolonged leucorrhea. In almost every instance I observed within a short time increase of appetite, improved nutrition, healthier color of the face, and increase of weight. I was surprised to learn how much more readily the Pepto-Mangan—Gude was taken than similar preparations, without ill effects even after protracted use.

To illustrate my remarks I will cite a few cases:

I will first report a case of chlorosis treated with this remedy, which was under constant observation. The patient, a schoolgirl aged 16, began to menstruate one year ago, but after appearing regularly for three periods the flow suddenly ceased, probably in consequence of mental over-exertion, and symptoms of chlorosis soon developed. The various preparations of iron were tried, but were either not well borne or excited so much disgust that they were discontinued by the capricious patient. A milk cure was prescribed, but followed for only a short time. When, however, I resorted to the Pepto-Mangan—Gude, I was surprised to find that the girl took it willingly and that it was well borne. She made a rapid recovery, and after the use of two bottles had regained her former healthy color, while her strength and menstruation returned.

CASE II.—A married lady, aged 24, had acquired—apparently of abortion at a very early period—an intense peri- and parametritis with exudation of the size of a child's head. The latter disappeared almost completely under suitable treatment and rest, so that only a slight induration was present in the parametrium after three weeks. Owing to the considerable anemia and loss of appetite, however, the patient recovered very slowly, and for this reason I ordered the Pepto-Mangan—Gude. A few days after its use the appetite reappeared, recovery ensued rapidly, and five weeks later her health was completely restored.

CASE III.—A married lady, aged 30, had suffered from leucorrhea due to catarrhal inflammation of the vagina for two years, and although the local trouble had been much relieved she continued pale and weak. As her chlorotic daughter at the time was taking the Pepto-Mangan—Gude with marked benefit, I advised her also to try this preparation. She followed my advice, and after fourteen days the weak, sluggish, and pale woman seemed as if transformed. She has since regained her former health.

These few cases, which were under continued observation, will confirm what has been said above regarding the manner of application and effect of the Pepto-Mangan—Gude. I regard it as superfluous to cite other cases, since a few closely observed cases teach more than a host of superficial observations.

On the ground of my experience I consider myself warranted in directing the attention of physicians to this remedy, and feel convinced that further trials will give equally favorable results. Even in cases where local treatment is necessary the Pepto-Mangan—Gude will prove a valuable auxiliary in our treatment.—*Allgemeine Weiner medizinische Zeitung*, xxxvi.

---

***NOTICE.—This JOURNAL and THE INTERNATIONAL JOURNAL OF SURGERY one year for $1,00 cash.***

### CATHETERS AND CYSTITIS.

BY R. N. MAYFIELD, M.D., NEW YORK,

Formerly President of the Colorado State Board of Medical Examiners
and Lecturer in Pathology and Clinical Medicine,
University of Colorado, etc.

It is well known that when it is necessary to use a catheter of usual construction—that is, with the ordinary fine perforations as an inlet thereunto—it does not work readily or satisfactorily, or subserve fully the results expected from it.

Examples of such unsatisfactory operations are seen where there is a good deal of mucus present in the bladder, such mucus being apt to surround or lie upon the end of the catheter, clogging or stopping the apertures thereof and preventing the ingress of fluids to be drawn off; again, when sediment or calcareous matter is present, it clogs, even sometimes filling in part or completely the apertures, with consequent failure of the catheter to fully perform its functions. Such failures are especially apt to happen in nearly, if not quite, all forms of chronic diseases of the bladder, and notably so in cystitis.

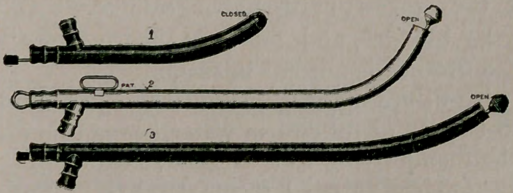

My object, therefore, is to present a catheter that is reliable and efficient in operation when the use of a catheter is indicated in all conditions and diseases of the bladder. In this instrument the danger of clogging or failure to perform its functions is obviated, and its interior may be readily made aseptic, and bits of mucus that usually clog an ordinary catheter may be readily drawn off.

This catheter is of very simple construction, being tubular, with the curve of an ordinary instrument, and open at the end for an inlet. For the closure of this open end, and for the easy insertion of the catheter, as well as for other purposes, a bulbous or rounded head is used, preferably solid, and attached to one end of a wire, passing through the body or tube and projecting at its rear or outlet end.

This construction forms a very efficient catheter having an area of opening so large as to greatly obviate the danger of clogging, for, if mucus should lodge against the open end, the working of the head back and forth upon its seat would cut away the obstructing bits of mucus and permit them to pass through the tube.

With this instrument there should be no hesitancy in using nitrate of silver, iodine, corrosive sublimate, carbolic acid, or hydrogen solutions in the bladder, as any of these solutions can be readily drawn off or neutralized, thus preventing poisoning from absorption, or preventing rupture from gases that form in the bladder.

Regarding the treatment of cystitis with the employment of this catheter, presuming that we have a typical case, with ropy, viscid, and tenacious mucus, the membrane thickened and possibly ulcerated, and in deep folds—"ribbed," as it were—we begin the treatment as follows:

1. Inject a quarter of a grain of cocain dissolved in a drachm of water into the membranous portion of the urethra.

2. Anoint the largest hard-rubber catheter that can be well passed into the bladder, and increase the size one number each week until the urethra is normal in size.

3. Begin with dilute hydrogen solutions—preferably hydrozone—one part to twenty of lukewarm water, using this solution freely, especially when employing the large size catheter. If the small size is used at the beginning, I recommend the use of only two or three ounces at a time until removed by the return flow. This can be repeated until the return flow is clear and not "foaming," which indicates that the bladder is aseptic.

4. Partly fill the bladder with the following solution: tincture of iodine compound, two drachms; chlorate of potassium, half a drachm; chloride of sodium, two drachms; warm water, eight ounces. Let it remain a minute or so and then remove. This treatment should be used once or twice a day.

Where I suspect extensive ulceration I recommend once a week the use of from ten to twenty grains of nitrate of silver to the ounce, and neutralize with chloride of sodium solutions.

This treatment carried out carefully will be satisfactory, as there is no remedy that will destroy bacteria, fetid mucus, or sacculated calcareous deposits like hydrogen.
